# Debriefing works: Successful retraction of misinformation following a fake news study

**DOI:** 10.1371/journal.pone.0280295

**Published:** 2023-01-20

**Authors:** Ciara M. Greene, Gillian Murphy

**Affiliations:** 1 School of Psychology, University College Dublin, Dublin, Ireland; 2 School of Applied Psychology, University College Cork, Cork, Ireland; The University of Queensland, AUSTRALIA

## Abstract

In recent years there has been an explosion of research on misinformation, often involving experiments where participants are presented with fake news stories and subsequently debriefed. In order to avoid potential harm to participants or society, it is imperative that we establish whether debriefing procedures remove any lasting influence of misinformation. In the current study, we followed up with 1547 participants one week after they had been exposed to fake news stories about COVID-19 and then provided with a detailed debriefing. False memories and beliefs for previously-seen fake stories declined from the original study, suggesting that the debrief was effective. Moreover, the debriefing resulted in reduced false memories and beliefs for novel fake stories, suggesting a broader impact on participants’ willingness to accept misinformation. Small effects of misinformation on planned health behaviours observed in the original study were also eliminated at follow-up. Our findings suggest that when a careful and thorough debriefing procedure is followed, researchers can safely and ethically conduct misinformation research on sensitive topics.

## Introduction

The increasing reliance of many on internet sources, including social media, for news and information has led to concerns about the prevalence of online misinformation. The term “fake news” came into use in 2016, and can be used to mean anything from intentionally disseminated falsehoods to inaccuracies in descriptions of news events [1]. The use of the term in academic research is disputed (with some preferring “false news” or “fabricated news”), but many researchers have settled on the definition provided by Lazer et al. [2], that fake news is “fabricated information that mimics news media content in form but not in organizational process or intent”. Discussions around the spread of fake news often allude to concerns that exposure to online misinformation might have significant consequences for public health or democratic institutions. This concern has been magnified with the onset of the COVID-19 pandemic and the associated “infodemic” [3–6]. As a result, a large body of research has investigated the effect of fake news and misinformation on participants’ memories, beliefs, attitudes and behaviours. The rise of this research field brings with it an obligation to establish whether experimentally-presented misinformation can be successfully retracted, and its influence eliminated.

### Consequences of misinformation exposure

Years of research have demonstrated that misinformation exposure can result in false or distorted memories; for example, when an eyewitness’s memory of a crime is influenced by a leading question [7], or when a participant is induced to remember a childhood event that never took place [8–10]. Similar observations have been made with respect to online misinformation, with various reports of false memories for fabricated events described in “fake news” articles [11–14]. However, probably the most oft-repeated concern with respect to fake news is the potential for misinformation to directly affect real-world behaviour. Exposure to misinformation in a laboratory setting can influence behaviour: for example, a body of research has examined the consequences of tricking participants into believing that they once became sick after eating a particular food [15–17]. In many cases, participants came to believe in or even remember this fictional event, and showed a subsequent unwillingness to eat that food when it was offered to them. A plethora of studies over the last decade have investigated participants’ belief in and willingness to share fake news (see [18] for a review); more recently, researchers have attempted to directly investigate its impact on behaviour. One study investigated effects of political misinformation exposure on voting behaviour, but the researchers were only able to measure effects at the municipal level by comparing the proportion of votes cast for populist parties [19]. The COVID-19 pandemic has awoken new interest in this topic, amid fears that misinformation might affect vaccine uptake or adherence to public health guidelines. Some research has suggested that anti-vaccination misinformation leads to vaccine hesitancy and reduced vaccination intentions [20, 21]. Others showed no effect of vaccine misinformation, even following multiple exposures to fake news headlines [22, 23]. In a large study of COVID-19 misinformation, Greene & Murphy recently reported that effects of a single exposure to a fabricated news story had small effects on subsequent behavioural intentions—for example, reading a story about privacy concerns with a forthcoming contact tracing app reduced intentions to download that app by about 5% [22]. What’s more, this study reported that participants who formed a false memory for the events described in the story experienced stronger effects on behaviour than those who simply saw the fake story but did not remember the events.

### Debunking and warnings

The potential for long-term harm arising from misinformation and fake news has led to the development of a variety of methods of reducing its impact. These methods generally fall into four categories: 1) specific debunkings or fact checks, in which a piece of misinformation to which participants have already been exposed is subsequently explained to be false (see [24] for a meta-analysis); 2) the use of specific warnings, in which false items are prefaced or accompanied by a warning label advising participants that the information they are about to read is inaccurate or disputed [25–28]; 3) efforts to ‘nudge’ news consumers into a more analytical frame of mind, for example by encouraging them to consider accuracy ([29, 30]), and 4) preventative measures in which researchers attempt to inoculate participants against future exposures to misinformation. This category includes gamified interventions designed to teach participants about online misinformation to help them detect it in future [31, 32], and generic warnings about the presence of misinformation, intended to increase participants’ tendency to monitor information more carefully. This last method is cheap and easy to implement, and is therefore the approach often used by governments or social media companies, who advise news consumers to “watch out for bad information” or “be media-smart” [33, 34]. Nevertheless, there is a stark lack of research addressing the effectiveness of these generic warnings. What research there is suggests that this approach may only be effective if it explicitly alludes to the information about to be presented. For example, Clayton et al. [35] presented participants with a general warning prior to exposure to misinformation that included the text, “you will be asked to evaluate the accuracy of some news headlines shared on social media. Although some of these stories may be true, others may be misleading”, and encouraged participants to be sceptical when reading the news headlines. Clayton et al. reported that this warning slightly reduced the perceived accuracy of the headlines. Greene & Murphy [22] went a step further and presented participants with generic warnings about misinformation that were not explicitly linked with the subsequently presented information, and found that they did not reduce acceptance of the misinformation—regardless of whether the warning was framed in positive or negative terms.

### Retracting misinformation: The role of debriefing

When misinformation is presented in an experimental context, researchers have an ethical obligation to retract that misinformation at the end of the procedure [36]. This is particularly important if the information has the potential to be harmful, for example, by suggesting that an alternative medicine might be an effective treatment for a disease. The extent to which misinformation can continue to exert effects on participants’ cognition or behaviour following debriefing is a pressing question. Within eyewitness memory research, a body of research has described the continued influence effect—the finding that misinformation that is presented to participants and subsequently withdrawn still colours or distorts their memories of the event (see [37] for a review). A similar observation has been made with respect to fake news or other forms of online misinformation and disinformation; researchers sometimes describe the information as “sticky” and difficult to eradicate. [38, 39]. In order for debriefing procedures to be effective at reducing belief and memory for misinformation, the debriefing must specifically debunk the misinformation provided; a general debrief is typically insufficient [40, 41]. Nevertheless, it was recently observed that less than a quarter of all misinformation papers published in the last six years reported providing a specific debriefing at the end of their experimental procedure [42]. In this context, it is important to consider the effects of debriefing on false memory as well as false belief, both in order to comply with our ethical obligation to leave participants as we found them [38], and because the presence of a memory may enhance subsequent attitudinal or behavioural change [15, 22]. It is, for example, possible that participants who form such false memories will experience persistent effects on behaviour that are resistant to debriefing.

One potential reason for the persistence or “stickiness” of misinformation is the so-called “sleeper effect”, whereby misinformation may be reported at higher rates following a delay, even if it has previously been debunked [43, 44]. This research suggests that a core memory of the original misinformation remains, while accompanying warnings, debunkings, or messages regarding source credibility fade away. As a result, misinformation that was initially accompanied by a warning or subsequently retracted might not be accepted by participants at initial testing, but may come to be believed or remembered over time. In the context of the COVID pandemic, and indeed other health-related topics, it is therefore critical to ascertain the long-term effects of misinformation exposure, and establish whether debunked misinformation continues to be believed, remembered, or acted upon.

Murphy et al. [45] recently reported a six-month follow-up of participants in a fake news study who were provided with a specific debrief at the end of the original study. Returning participants were less likely to report a false memory for a story they had previously been exposed to than new participants, who had not taken part in the original study, and were also less likely to form a false memory for a novel fake story. This provided strong support for the suggestion that debriefing is effective in reducing false memory for the specific misinformation provided, and may have a protective effect against future misinformation. The interval between debriefing and follow-up in that study was rather long, however. In the absence of reminders or post-event information, memories tend to decay over time [46]. Thus, the effects of misinformation may have simply faded over the course of a six-month period, but continued to have an influence for some time after debriefing. Indeed, in the context of a constantly shifting information landscape, such as that accompanying the COVID-19 pandemic, it may be more appropriate to focus on potential effects over a shorter timescale. For example, a researcher may have a valid concern that exposing a participant to misinformation about vaccination might affect their decision to get vaccinated in the following days or weeks. It remains to be seen whether debriefing is effective in reducing misinformation acceptance in the shorter term.

### Memory vs. belief

When evaluating the impact of misinformation and the effectiveness of debriefing, it is important to consider the distinction between false memory and false belief. It has previously been suggested that many reports of false memory in the literature may in fact reflect false belief—instances where the participant believes that the event in question took place, but does not have a clear memory of it [47, 48]. Recent evidence has suggested that memory and belief may have discriminable effects on subsequent behavioural intentions; for example, participants who were given a false suggestion that they had previously become ill after eating a certain food are more likely to change their behaviour if they *believe* the false information than if they simply *remember* it [16, 49]–the difference being recalling a memory of the event and actually believing that it truly took place. This can be ameliorated during data collection by explicitly distinguishing between memories and beliefs, for example by asking participants to indicate whether they clearly remember seeing or hearing about the event, or simply believe that it happened (e.g. [11, 12, 50]). Similarly, following a debriefing, it is important to distinguish whether participants still *believe in* or *remember encountering* the debunked information. It is not uncommon for people to retain a memory of an event even after they come to believe it never happened; for example, many people remember seeing Santa Claus coming down the chimney as a child, but as an adult no longer believe that to be a veridical experience. These “nonbelieved memories” [51, 52] may be expected to have less impact on our future behaviour; for example, you are unlikely to leave cookies out for Santa on Christmas Eve if you don’t believe he really exists, regardless of your childhood memories. Similarly, participants may retain the *memory* of having previously encountered the events described in a fake news story, but subsequently come to understand that the events never took place and should not affect their decision making. Of note however, recent work by Burnell and colleagues [53] suggests that retracted memories can still serve both helpful and harmful functions for individuals—for example, by influencing thinking or social cohesion.

### The present study

In the present study, we report data from a one-week follow-up of participants who engaged in a study of COVID-19 misinformation, and were subsequently debriefed. In the original study, described in Greene & Murphy [22], participants were randomly assigned to receive a warning about misinformation or to a control condition, and were later exposed to a selection of fabricated news headlines related to the COVID pandemic. Participants were asked to report whether they remembered the events described in the headline, how truthful they believed the story to be, and how likely they were to engage in a series of health behaviours linked with the fabricated stories.

In this follow-up study, we address the following preregistered research questions:

1. Do participants from a previous COVID-19 fake news study continue to report false memories or beliefs one week later, despite having been debriefed after the first study?
2. Does previous exposure to COVID-19 misinformation have persistent effects on planned health behaviours following debriefing?
3. Does previous exposure to a general (non-specific) warning about misinformation moderate effects of misinformation on memories and planned behaviours after one week?

From a purely experimental perspective, one might wish to compare responses from debriefed and undebriefed participants. However, the timing of this experiment (conducted in mid-2020, at the height of the COVID pandemic and in the midst of an ‘infodemic’) meant that we were very reluctant to have participants leave the study without correcting misinformation. We therefore designed the experiment such that participants were randomly assigned to view half of the fake news items in the original study and the other half at follow-up, permitting a direct comparison of the effect of novel vs. previously seen misinformation.

## Materials and methods

### Preregistration

The hypotheses and analysis plan for this study were preregistered at https://aspredicted.org/PMV_N5Q. Ethical approval for this study was granted by the Human Research Ethics Committee at University College Dublin.

### Participants

Participants were recruited for the original study [22] via an article in the Irish news website TheJournal.ie in May 2020. Of the 4,228 participants who completed the original study, 2,282 provided a valid email address for follow-up, and 1,738 completed the follow-up study. In line with our preregistration, 191 of these were excluded for failing attention checks or admitting to having used a search engine to look up answers to the questions. The final sample in the follow-up study comprised 1,547 participants with a mean age of 48.48 years (SD = 12.75), and included 474 (30.64%) males, 1,070 (69.17%) females and 3 participants (0.19%) who selected ‘other’ or preferred not to indicate their gender.

### Materials and procedure

**Fig 1:**
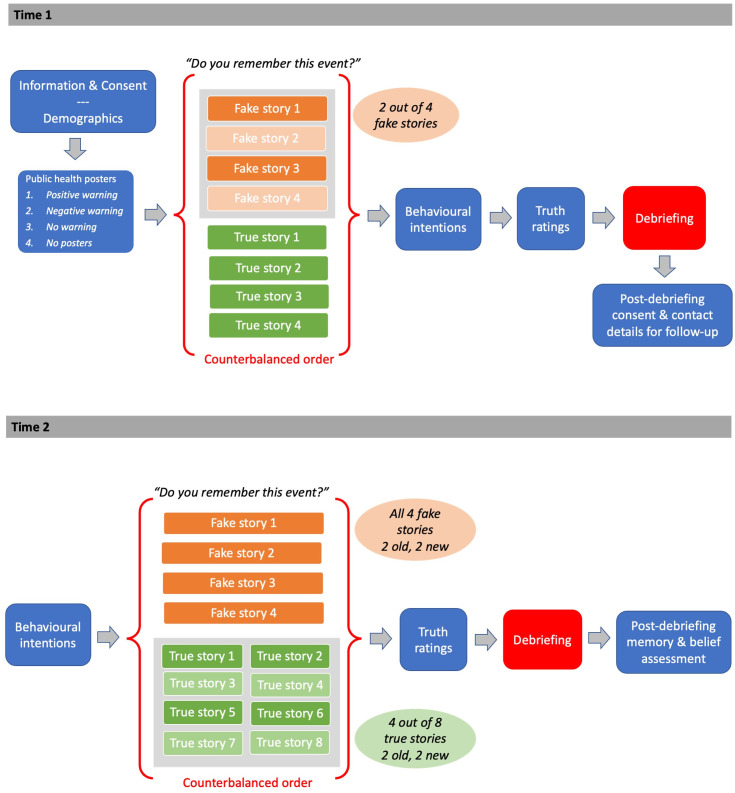
Schematic of experimental procedure at Time 1 and Time 2. Note: A series of measures assessing individual differences in cognitive ability and knowledge about COVID-19 were also collected at Time 1, with their presentation counterbalanced with the measures described here. These measures are not relevant to the present paper, and so are omitted from this schematic in the interests of clarity.

A schematic of the experimental procedure is provided in Fig 1.

#### Original study (Time 1)

The materials and procedure for the original study are described in detail in Greene & Murphy [22], and all materials are available at https://osf.io/mfnb4/. Participants provided written consent to participate, but were not initially told that the study was investigating misinformation. In order to manipulate the presence of a warning, participants were first presented with a series of public health messages, similar in format to posters produced by the Irish Health Service Executive (HSE) during the COVID-19 pandemic. Mixed in among messages about social distancing and cough etiquette, half of the participants in the sample were randomly assigned to a warning condition in which they were exposed to a positively or negatively-framed warning about misinformation (positive framing: “Not all news stories we read are accurate. During the COVID-19 crisis, it’s important that we all play our part in society by thinking carefully about the stories we read and share. Think before you share and keep your loved ones safe!”. Negative framing: “Not all news stories we read are accurate. Sharing stories that may not be true is irresponsible, and puts us all in danger during the COVID-19 crisis. Think before you share and keep your loved ones safe!”). The remaining participants were either presented with the other public health messages, without the misinformation message, or were in a pure control group that received no health messages at all. The warning conditions had no effect on any outcome variable in the original study (see [22] for a discussion).

Participants were then presented with six news stories about the COVID-19 pandemic, including four true stories and a randomly selected two out of four fake stories. Each story consisted of a short text description, accompanied by an illustrative but non-probative photograph. The four fake stories were fabricated for this study, and read as follows:

1. “New research from Harvard University shows that the chemical in chilli peppers that causes the "hot" sensation in your mouth reduces the replication rate of coronaviruses. The researchers are currently investigating whether adding more spicy foods to your diet could help combat COVID-19” [Accompanying photograph: a pile of red chilli peppers].
2. “A whistleblower report from a leading pharmaceutical company was leaked to the Guardian newspaper in April. The report stated that the coronavirus vaccine being developed by the company causes a high rate of complications, but that these concerns were being disregarded in favour of releasing the vaccine quickly” [Accompanying photograph: a close-up of a hypodermic needle being inserted into a patient’s arm].
3. “A study conducted in University College London found that those who drank more than three cups of coffee per day were less likely to suffer from severe Coronavirus symptoms. Researchers said they were conducting follow-up studies to better understand the links between caffeine and the immune system” [Accompanying photograph: a close-up of a steaming cup of coffee].
4. “The programming team who designed the HSE app to support coronavirus contact-tracing were found to have previously worked with Cambridge Analytica, raising concerns about citizen’s data privacy. The app is designed to monitor people’s movements in order to support the government’s contact-tracing initiative” [Accompanying photograph: A close-up of a smartphone with COVID-19 imagery, overlaid on an illustration of the coronavirus organism].

After each story, participants were asked whether they remembered the events described in the story, and could select from the following options: “I have a clear memory of seeing/hearing about this”, “I have a vague memory of this event occurring”, “I don’t have a memory of this, but it feels familiar”, “I remember this differently” or “I don’t remember this”. In line with our preregistration, participants who selected one of the first two options were deemed to have a memory for the event. Thus, “memory” was defined as either a specific or non-specific memory of the event having occurred. Participants were then asked to indicate the source of their memory from a list (e.g., television, newspaper, radio, online news website etc.). Finally, participants were asked to indicate how they had felt about the events in the story at the time, via an open text box.

Participants were then asked to reflect on their intention to engage in a series of health behaviours over the next several months, and to rate their agreement with statements about the behaviours on a scale from 1 (strongly disagree) to 7 (strongly agree). The four critical statements associated with the fake stories read, “I intend to eat more spicy food”, I intend to drink more coffee”, “I intend to get a COVID-19 vaccine, once it becomes available” and “I intend to download the HSE contact-tracing app, once available”. Six filler statements were also included, and addressed intentions regarding other health behaviours, including intention to get more sleep, reduce screen time and get a flu vaccine. The full text of the behavioural intention statements may be seen in online materials at https://osf.io/mfnb4/.

Finally, participants were informed that some of the stories they had seen may not have been true. They were presented with a thumbnail image of each of the six stories that they had earlier been shown, and were asked to rate the truthfulness of each story on a scale from 0 (definitely not true) to 100 (definitely true).

Participants in the original study also completed a series of other measures, assessing individual differences in analytical reasoning as well as knowledge of and engagement with the topic of COVID-19. These measures, the presentation of which was counterbalanced with the other questions described above, are not relevant to the follow-up study and will not be discussed further here. Interested readers may refer to Greene & Murphy [48] for an analysis of the effects of these individual differences on susceptibility to false memories.

#### Debriefing

Immediately after completion of the study, participants were fully debriefed, following the debriefing procedure described in Murphy et al. [45]. The two fake stories that the participant had seen were then presented again, accompanied by an explanation that the story was not true but had in fact been fabricated by the researchers. This message was reinforced with true information about the topic of the fake story (e.g. “This story is not true. There is no known association between the HSE contact tracing app and Cambridge Analytica”). A full debriefing statement was then provided, in which participants were informed about the true purpose of the study and the ease with which false memories and false beliefs may form. Participants were then asked to re-consent to the inclusion of their data in the study and to provide an email address if they were willing to be contacted for a follow-up study.

#### Follow-up study (Time 2)

One week after completion of the original study, participants who had provided an email address were contacted and asked to complete a five-minute follow-up survey via Qualtrics. This study began with a repetition of the ten behavioural intention statements from Time 1, presented in random order. Participants were then presented with eight news stories, presented in random order. These included the four fake news stories from Time 1 –two of which the participants had previously seen, and two of which were novel to them—and four true stories, including two previously seen stories and two novel ones. After each story, participants were asked to report whether they remembered the events described in the story, using the same options from Time 1, and to indicate where they had previously encountered the story using an open text box. As at Time 1, a false memory was defined as a participant selecting the response, “I have a clear memory of seeing/hearing about this” or “I have a vague memory of this event occurring” for one of the fake stories. In order to ensure that participants were not simply reporting having seen the story in part 1 of this study, we reviewed the responses in the open text box. Responses in which the participants indicated that they had previously seen the story at an earlier stage of this study (e.g., "last survey" or “you presented it in the study last week”) were not counted as false memories. Responses in which the participant reported remembering the event and either provided a non-study source or indicated that they did not remember the source were coded as memories; for example, responses such as, “on line somewhere”, “social media” or “heard a friend talking about it” indicated non-study sources for the fabricated information. These responses are therefore taken to indicate genuine false memories for the events depicted in the fake stories.

After viewing all eight stories, participants were told that some of the stories they had seen may not have been true and were asked to rate the truthfulness of each story on a scale from 0 (definitely not true) to 100 (definitely true). Participants were then told, “Some participants were shown the same stories in both parts of this study. Please select any story below that you believe was shown to you last week in Part 1 of this study”. This was followed by a debriefing in which participants were shown all four fabricated stories along with an explanation that the story had been fabricated and some information about the truth of the story.

Finally, participants were told that people may sometimes retain a memory of an event, even after they have learned that it never happened. Participants were asked to consider whether they currently *believed* or *remembered* the events described in each of the fake stories, selecting from the options, “I have a memory of this, AND I believe it happened”, “I have a memory of this, BUT I don’t believe it happened”, I don’t have a memory of this, BUT I believe it happened” and “I don’t have a memory of this, AND I don’t believe it happened”. Before leaving the study, participants were presented with a final debriefing document and links to reliable sources for information about the COVID-19 pandemic.

## Results

### Persistence of misinformation effects: False memory

**Fig 2:**
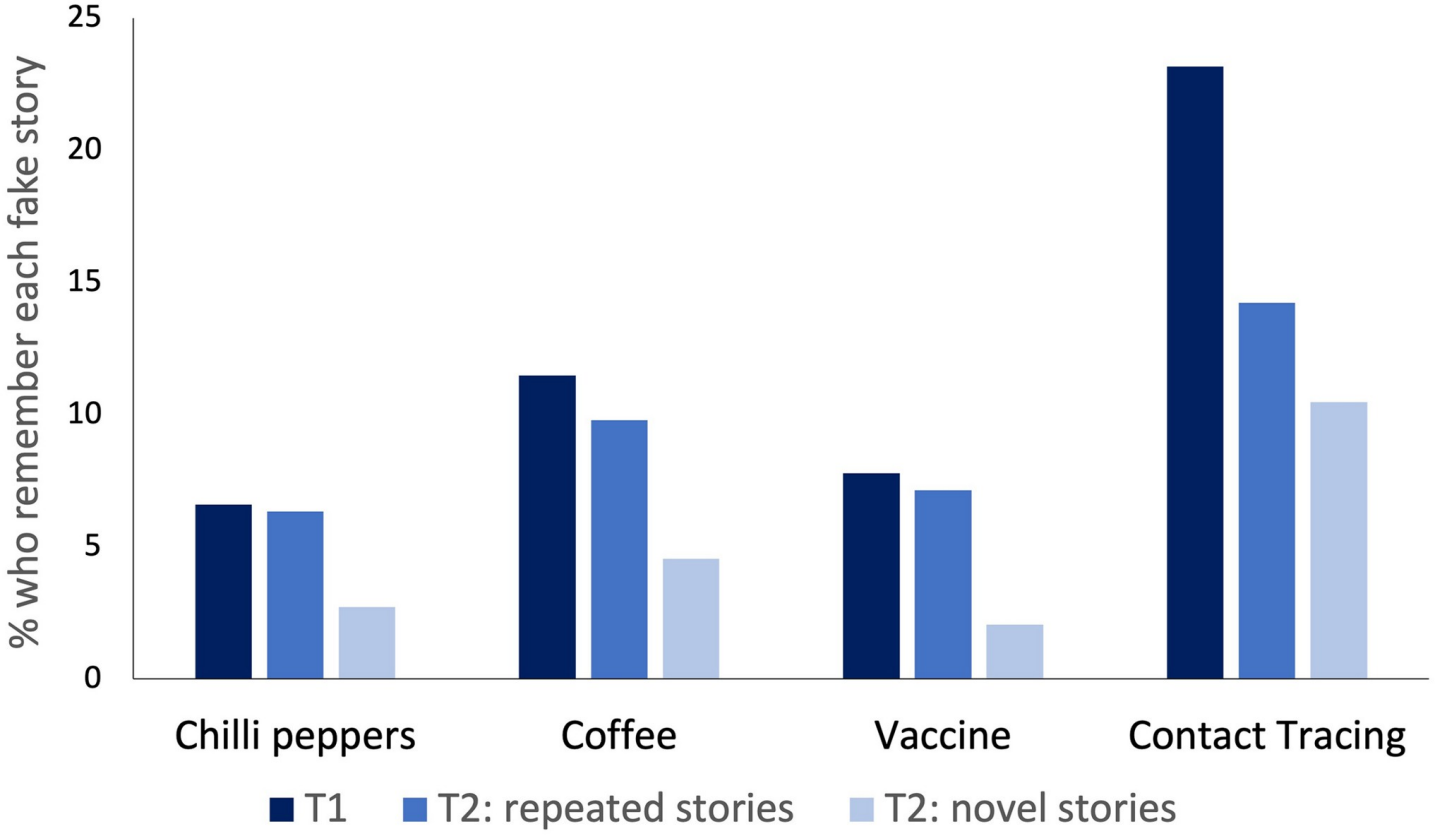
Percentage of participants who reported a false memory for each fake story in the original study (T1) and at follow-up (T2). T2 false memory rates are displayed separately for stories to which the participant had previously been exposed (repeated stories, seen at T1 and T2) and stories to which the participant had not previously been exposed (novel stories, seen at T2 only).

Fig 2 depicts the percentage of participants who reported a false memory for each fake story in the original study (Time 1) and at follow-up (Time 2). The overall rate of false memory declined from 12% at Time 1 (M = 0.24 out of 2 fake stories, SD = 0.48) to 7.25% at Time 2 (M = 0.29 out of 4 fake stories, SD = 0.59; (t(1546) = 7.77, p < .001, *d* = 0.20). As Fig 1 shows, false memories were reduced for all four stories, however this only reached statistical significance (p < .001) for the coffee and contact tracing stories, which had resulted in the highest number of initial memories. The reduction in false memory rate was statistically significant for both repeated stories (to which the participant had previously been exposed, t(1546) = 3.85, p < .001, *d* = 0.1) and novel stories (to which the participant had *not* previously been exposed, t(1546) = 10.26, p < .001, *d* = 0.26). This suggests that the effect of debriefing generalised to subsequent false memories for new stories that had not previously been presented or retracted.

For each of the four fake stories, we compared the frequency of false memories at follow-up between participants who saw the story for the first time (novel stories) and participants who had previously seen the story at the Time 1 (repeated stories). Across all items, participants were significantly more likely to report a false memory for a repeated story than for a novel story (see Table 1 for details). The low rate of false memories for novel stories suggests that participating in the original study (and being debriefed) made participants less accepting of additional news items presented to them during the follow-up assessment.

**Table 1:** Percentage of participants who reported a false memory at follow-up for novel and repeated stories.

	False memory rates		N	Comparison
	Novel Stories	Repeated Stories		
Chilli peppers	2.72%	6.33%	1547	*χ*^2^(1) = 11.69, p < .001, *V* = 0.09
Coffee	4.54%	9.79%	1547	*χ*^2^(1) = 16.03, p < .001, *V* = 0.10
Vaccine	2.06%	7.13%	1547	*χ*^2^(1) = 22.72, p < .001, *V* = 0.12
Contact tracing	10.47%	14.23%	1547	*χ*^2^(1) = 5.07, p = .02, *V* = 0.06

Note: Our preregistration specified the use of McNemar’s tests for these comparisons, however as the novel and repeated conditions were manipulated between groups, the more appropriate chi-squared test was used instead.

Participants were also more likely to report a false memory for a story at follow-up if they had originally reported remembering that story at Time 1. For example, 25.53% of participants who originally remembered the chilli peppers story reported a memory for that story at follow up, compared with 5.12% of participants who had not reported a memory for the story at Time 1). Similar results were observed for the other stories (see Table 2). Those who had remembered the stories at T1 also tended to find them more believable at Time 2; see Table 2 for details.

**Table 2:** False memories and truthfulness ratings at Time 2 for stories that were initially remembered or not remembered at Time 1.

Story	Memory report at Time 1		Comparison
	Memory	No memory	
*False memories at Time 2*	% (N)	% (N)	
Chilli peppers	25.53% (12)	5.12% (37)	*χ*^2^(1) = 27.23, p < .001, N = 774, *V* = 0.19
Coffee	23.6% (21)	8.01% (55)	*χ*^2^(1) = 21.68, p < .001, N = 776, *V* = 0.17
Vaccine	21.67% (13)	5.91% (42)	*χ*^2^(1) = 20.74, p < .001, N = 771, *V* = 0.16
Contact tracing	22.35% (40)	11.78% (70)	*χ*^2^(1) = 12.57, p < .001, N = 773, *V* = 0.13
*Truthfulness rating at Time 2*	M (SD)	M (SD)	
Chilli peppers	11.36 (22.65)	7.89 (17.12)	t(53.09) = 1.06, p = .29, *d* = 0.17
Coffee	20.24 (27.37)	12.40 (22.07)	t(95.92) = 2.51, p = .01, *d* = 0.32
Vaccine	35.44 (35.11)	16.41 (24.89)	t(25.52) = 2.51, p < .001, *d* = 0.63
Contact tracing	43.68 (38.53)	32.96 (37.15)	t(269.74) = 3.21, p = .01, *d* = 0.28

Note: Between groups comparison of truthfulness data is conducted using Welch’s t-test to account for unequal groups

### Persistence of misinformation effects: False belief

**Fig 3:**
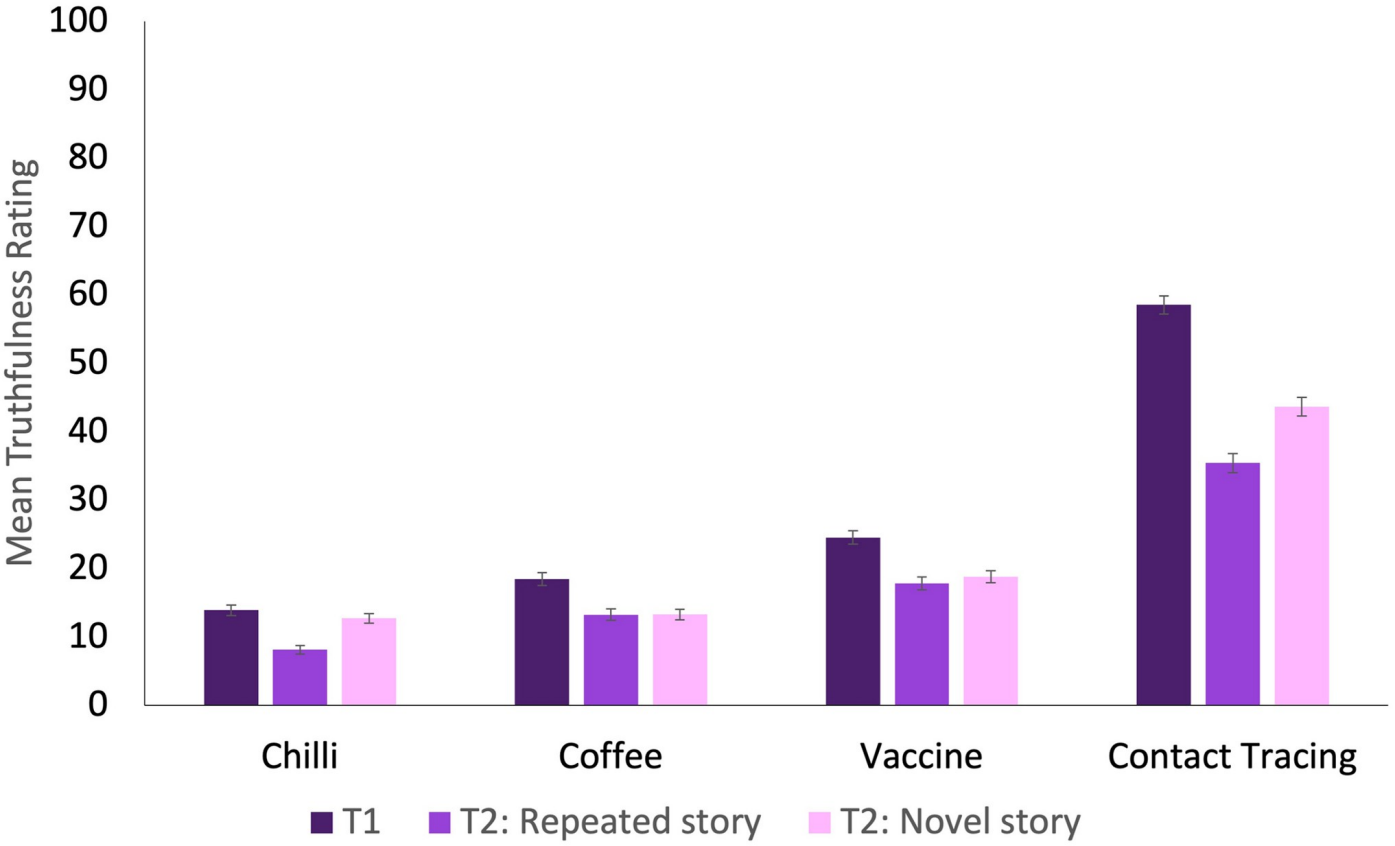
Mean truthfulness rating for each fake story in the original study (T1) and at follow-up (T2), presented separately for novel and repeated stories. Error bars represent standard errors of the mean.

As with the false memory data, the mean truthfulness rating for the fake stories declined from Time 1 (M = 28.93, SD = 24.37) to Time 2 (M = 20.60, SD = 17.67; t(1510) = 11.98, p < .001, *d* = 0.31), indicating that participants were less inclined to believe the fabricated stories at follow-up. This difference was significant for both novel (t(1483) = 8.01, p < .001, *d* = 0.21) and repeated stories (t(1483) = 14.15, p < .001, *d* = 0.37). At Time 2, participants judged novel stories (M = 22.06, SD = 22.05) to be more truthful than repeated stories (M = 18.88, SD = 22.87; t(1498) = 3.98, p < .001, *d* = 0.10; see Fig 3). This stands in contrast to the finding that higher rates of false memories were observed for repeated stories than for truthful stories. This suggests either that participants were in fact remembering the fabricated stories from part 1 of the present study—but did not list our previous study as the source of the memory—or that the reported memories include some proportion of non-believed memories, which participants no longer believe to be true.

In line with our preregistration, participants were deemed to have believed a story at Time 1 if they rated its truthfulness as greater than 50 on a 1–100 scale. By this metric, fewer than 10% of participants believed the chilli peppers, coffee or vaccine stories to be true, with approximately 28% of participants believing the contact tracing story to be true. Using this categorisation, we compared false memories at follow-up between participants who believed the story at Time 1, and those who did not. Participants who originally believed the story were more likely to retain a false memory at follow-up for the coffee story (remembered by 20.65% of participants who originally believed the story, vs. 8.44% of those who did not; *χ*^2^(1) = 13.43, p < .001, N = 744, *V* = 0.13), vaccine story (remembered by 11.57% of believers vs. 6.34% of non-believers; *χ*^2^(1) = 4.10, p = 0.04, N = 720, *V* = 0.08) and contact tracing story (remembered by 17.16% of believers vs. 9.12% of non-believers; *χ*^2^(1) = 9.29, p = .002, N = 728, *V* = 0.11). No significant effect was observed for the chilli peppers story (remembered by 2.2% of believers and 6.6% of non-believers; *χ*^2^(1) = 1.37, p = .24, N = 741, *V* = 0.04).

To further investigate his question, we analysed post-debriefing reports of memories and beliefs. Following the second debriefing at the end of the follow-up study, participants were asked to report whether they still retained a belief or a memory for each of the fabricated stories. This question was completed by 1501 participants. As shown in Table 3, the most common response for each story was “I don’t have a memory of this, AND I don’t believe it happened”. Nevertheless, a substantial proportion of participants reported a non-believed memory—that is, they reported still retaining a memory of having previously encountered the story prior to taking part in our study, while no longer believing the events depicted in the story actually occurred. Examination of this table indicates that the contact tracing story was the “stickiest” item, with 11.52% of participants still reporting a memory and belief in the story following debriefing. This suggests that some individuals may be resistant to correction of particularly convincing fake news items, particularly if they have formed a false memory of having encountered the story before.

**Table 3:** Number and percentage of participants who report remembering and/or believing each fake story following the second debriefing at the end of the follow-up survey (Time 2).

Story	Memory and belief	Non-believed memory	Belief, no memory	No memory or belief	Total memories	Total beliefs
Chilli peppers	27 (1.8%)	304 (20.25%)	44 (2.93%)	1126 (75.02%)	331 (22.05%)	71 (4.73%)
Coffee	51 (3.39%)	337 (22.42%)	57 (3.79%)	1058 (70.39%)	388 (25.81%)	108 (7.18%)
Vaccine	36 (2.4%)	265 (17.65%)	74 (4.93%)	1126 (75.02%)	301 (20.05%)	110 (7.33%)
Contact tracing	173 (11.52%)	370 (24.63%)	151 (10.05%)	808 (53.79%)	543 (36.15%)	324 (21.57%)

### Effect of warnings on misinformation acceptance

A principle aim of this study was to investigate the effects of general warnings about misinformation at Time 1 on continued acceptance of misinformation at Time 2. As noted in the Methods section, participants were randomly assigned to one of four warning conditions: positive framed warning, negatively framed warning, no misinformation warning and no warning posters. In line with our preregistration, misinformation acceptance was assessed using two measures:

1. d’, a measure of participants’ ability to discriminate between memories for true and fake stories. The d’ measure derives from signal detection theory [54], which describes the behaviour of an observer searching for a signal in a noisy field. In the context of memory construction, a feeling of familiarity with an event one has experienced before may be considered to be the ‘signal’. By the same token, a feeling of familiarity with a never-before-experienced item can be considered a false alarm. Individuals with better discrimination abilities will be better able to identify the signal (true memories) against the background of noise (false memories). See [11] for further discussion of this issue. d’ was calculated as the difference between the standardised rate of hits and false alarms, where ‘hits’ were defined as memories for the true stories and ‘false alarms’ were defined as memories for the fake stories. d’ was computed with respect to all stories at Time 1, and with respect to novel stories only at Time 2.
2. Average truthfulness ratings, computed separately for true and fake stories. As with the calculation of d’, truthfulness ratings encompassed all stories from Time 1, and novel stories only at Time 2.

**Fig 4:**
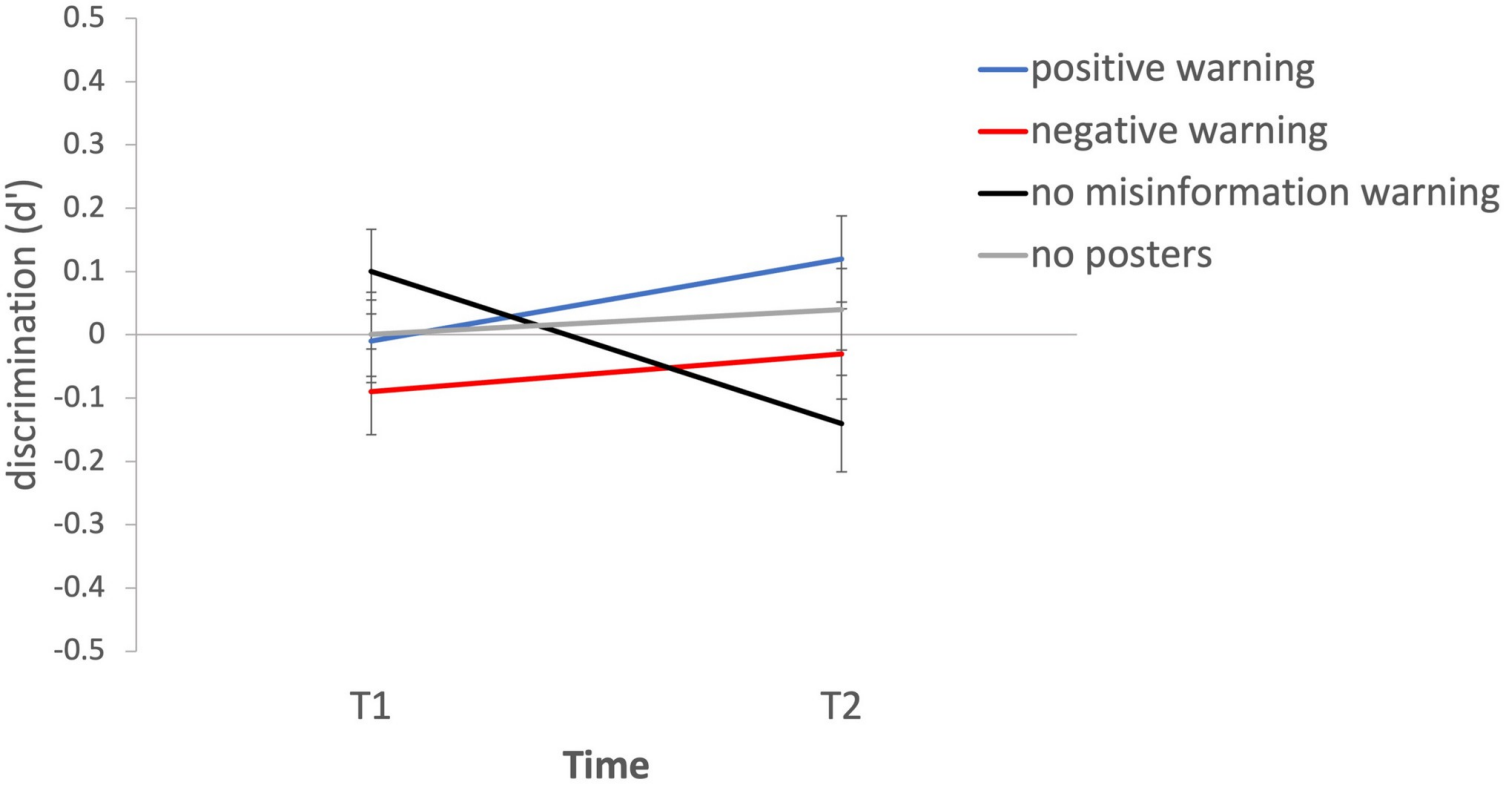
Mean d’ values as a function of time and warning condition, indicating participants’ ability to discriminate between memories for true and fake stories. Error bars represent standard error of the mean.

A two-way ANOVA examined the effects of warning condition and time (T1, T2) on d’. There was no main effect of time (F(1,1543) = 0.002, p = .97) or warning condition (F(3,1543) = 0.93, p = .42), however a significant interaction effect was observed (F(3,1543) = 3.09, p = .03, η_p_^2^ = 0.006). As depicted in Fig 4, discrimination ability increased slightly from T1 to T2 in the positive warning condition, and decreased from T1 to T2 in the no misinformation warnings condition, however post hoc Tukey tests did not indicate any significant pairwise comparisons. The fact that a slight difference from T1 to T2 is observed in the no warnings condition, but not in the no posters condition, coupled with the very small effect sizes and lack of significant pairwise differences, suggests that this interaction may be spurious. Interested readers may also find an analysis of the effects of time and warning condition on true memory rate (memories reported for true stories) in the S1 File; no significant effects were observed.

A three-way ANOVA assessed the effects of story type (true/fake), warning condition and time on average truthfulness rating. As would be expected, a significant effect of story type was observed, such that true stories were rated as more truthful than fake stories (True: M = 71.16, SE = 0.44; Fake: M = 25.30, SE = 0.44; F(1,1468) = 5859.75, p < .001, η_p_^2^ = 0.80). There was no main effect of time or warning condition, and no interaction between warning condition and any other variable (see S1 File for full details of this analysis).

In sum, neither the presence or absence of a general misinformation warning nor the type of warning provided had a consistent effect on participants’ ability to discriminate between true and fake news.

### Planned health behaviours

In order to evaluate the potential long-term effects of exposure to misinformation on health behaviours, we conducted four two-way ANOVAs examining the effect of exposure to a fake news story at Time 1 and warning condition on the related behavioural intention at Time 2. A sleeper effect would be denoted by an increased intention to engage in the behaviour suggested by the fabricated story after a one-week delay, while a reduced intention to engage in that behaviour would indicate an effect of the debriefing procedure.

**Fig 5:**
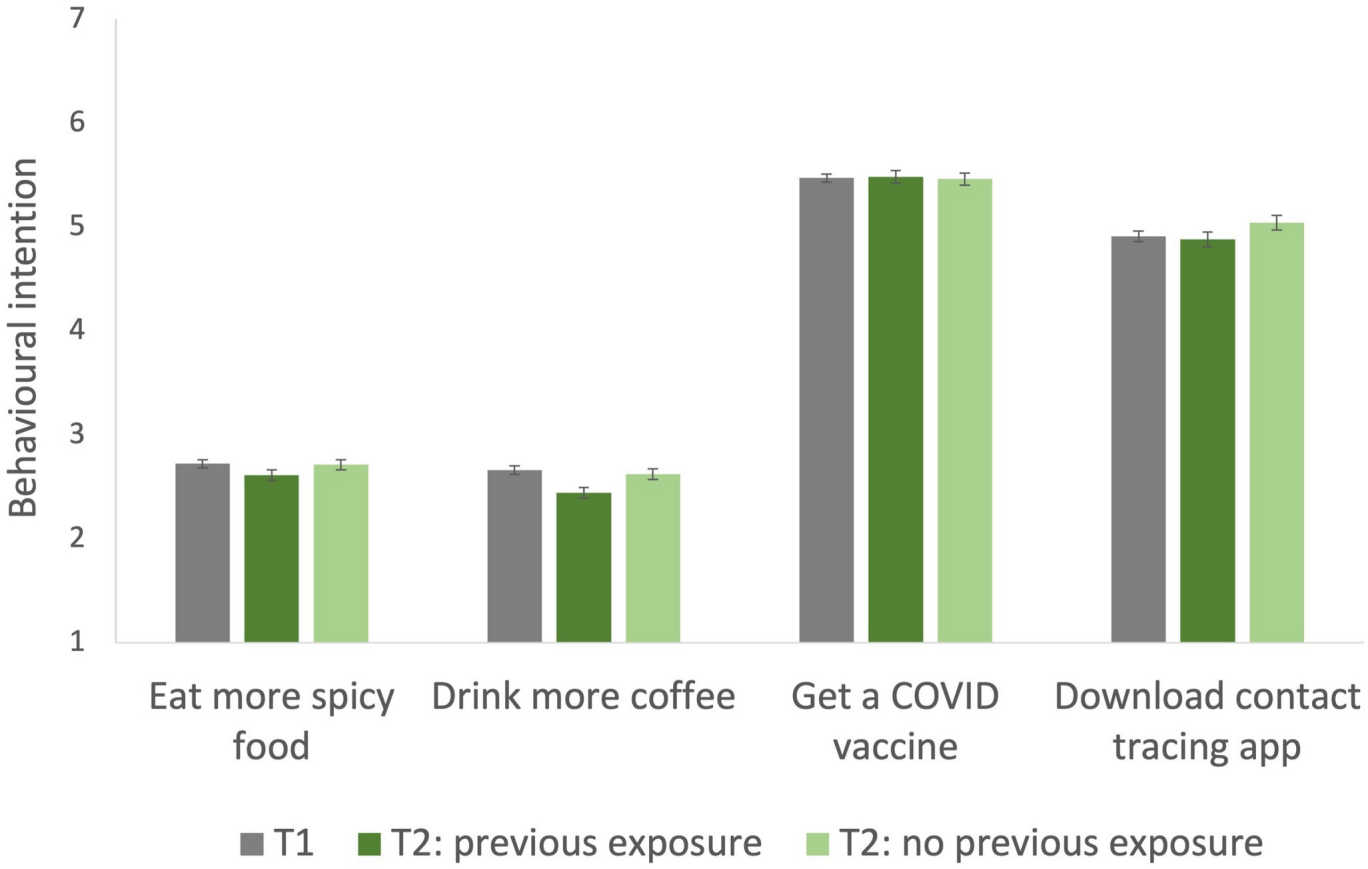
Mean behavioural intention scores for the targeted behaviours at Time 2, separately for participants who were or were not exposed to the associated misinformation at Time 1. Time 1 data for exposed participants is provided for comparison. 1 = strongly disagree [that I intend to engage in the targeted behaviour], 7 = strongly agree. Error bars represent standard error of the mean.

A significant effect was observed with regard to the coffee story, such that participants who were exposed to that story at Time 1 (and debriefed) were *less* likely than participants who had never seen that story before to report an intention to drink more coffee (previous exposure: M = 2.44, SE = 0.05; novel story: M = 2.62, SE = 0.05; F(1, 1529) = 6.25, p = .01, η_p_^2^ = .004). This suggests a reduced intention to act on the misinformation contained in the story following debriefing, but the effect is very small. No other significant effects of exposure to fake news were observed on behavioural intentions at follow-up (chilli peppers: F(1,1532) = 1.95, p = 0.16, η_p_^2^ = .001; vaccine: F(1,1529) = 0.05, p = 0.83, η_p_^2^ < .001; contact tracing: F(1,1534) = 1.59, p = 0.19, η_p_^2^ = .003). See Fig 5 for details. As in our original study, there were no significant effects of warning condition (all p’s < .3).

To examine effects of false memories for misinformation on health behaviour, we conducted an additional set of two-way ANOVAs among participants who were exposed to each fake story at Time 1. The independent variables in these analyses were original response to each story at Time 1 (remembered/did not remember) and warning condition, while the DV is intention to change the associated behaviour. No significant effects were observed; after one week, participants who had originally formed a false memory for the fake news story were no more likely to engage in the proposed behaviour than participants who saw (but did not remember) the fake story. Full details of these analyses may be found in S1 File.

## Discussion

This study aimed to determine the fate of debunked misinformation, one week following a detailed debriefing. Our first research question asked whether participants would continue to report false memories or beliefs one week later, despite being debriefed after the first study. Both false memory rates and belief in the fake stories declined between the original study (Time 1) and the follow-up study (Time 2), suggesting that the debriefing was effective in retracting both false memory and false belief. People who initially reported a memory for a given story were more likely to remember it at Time 2, and considered it to be more truthful, compared with people who saw the story but didn’t form a false memory of it.

Analysis of false memories revealed that participants were more likely to report remembering repeated stories (which had been presented at Time 1 and Time 2) than novel stories (presented at Time 2 only). In contrast, participants found the novel stories to be more believable than the repeated, debunked ones. This apparent contradiction was resolved by an examination of participants’ explicit statements about whether they *believed* or *remembered* each of the fake stories at the end of the procedure. In line with previous research [49], the majority of persistent memories were thus identified as non-believed memories, suggesting that debriefing works well to reduce belief in a story, even if participants still retain the memory of it. Interestingly, these rates of non-believed memories are higher than the false memory rates obtained prior to the second debriefing, when participants were simply asked to report whether they had a memory for each news story. This may suggest that some participants intentionally discounted memories that they no longer believed to be true, and did not consider them to be ‘real’ memories. Importantly, false memory and belief were significantly reduced relative to Time 1 for both repeated and novel stories, providing evidence that the debriefing led participants to be more suspicious and less likely to take new misinformation at face value. It is however important to note that the follow-up study was clearly associated with the original study; future research may wish to examine the effects of the debriefing on subsequent exposure to misinformation from other sources.

Our second research question asked whether previous exposure to COVID-19 misinformation would have persistent effects on planned health behaviours following debriefing. Here, we were interested in the effectiveness of our debriefing and the potential for sleeper effects, whereby the effect of misinformation may be magnified after a delay [44, 55]. Analysis of behavioural intentions revealed no evidence of sleeper effects: participants who were exposed to a particular piece of misinformation at Time 1 were no more likely to report an intention to engage in that behaviour after one week compared with participants encountering the information for the first time at Time 2. On the contrary, a trend suggesting reduced intentions among those participants who were exposed and debriefed was observed, though the effect was only significant for the coffee story. This is suggestive of a protective effect from the debriefing, whereby having taken part in the initial study rendered participants slightly less likely to act on the information contained in the fake stories. Overall, however, behavioural intentions changed very little in the one-week interval between the original study and follow-up. In our initial study [22], intentions to engage in some of the targeted health behaviours was increased among participants who reported a memory for the associated fake story. In this follow-up data, that effect is no longer observed, and participants who remembered the fabricated events were no more likely to engage in the behaviour. Thus, the debriefing was effective in retracting effects of misinformation on future behaviour among participants who initially found it very convincing, but all behavioural effects were weak and inconsistent across stories.

Our final question asked whether previous exposure to warnings about misinformation would moderate effects of misinformation on memories and planned behaviours after one week. In line with our original study, no effects of warnings were observed. We therefore conclude that non-specific warnings about the presence of misinformation have no impact on response to that misinformation either immediately or after a delay.

The findings from this study are broadly in line with those of Murphy et al. (2020), and indicated that a detailed post-experimental debriefing can effectively undo any potential harm caused by misinformation, at intervals from one week (present study) to six months [45]. Future research may wish to delay debriefing of a control group in order to directly compare the long-term effects of different types of debriefing. As noted above, this was not possible in the present study due to ethical considerations pertaining to the risks associated with failing to debrief participants during a public health crisis. Nevertheless, we conclude that researchers who provide a careful and detailed post-experimental debriefing may engage in misinformation research on sensitive or high-risk topics, secure in the knowledge that they are meeting their ethical obligations to retract misinformation and avoid harm to participants.

## Supporting information

S1 File (DOCX)
