# Supplementary material for: Debriefing works: Successful retraction of misinformation following a fake news study
Source: PLoS One. 2023 Jan 20;18(1):e0280295. doi: 10.1371/journal.pone.0280295 (PMC9858761; doi:10.1371/journal.pone.0280295)
Supplement: S1 File — (DOCX) [file pone.0280295.s001.docx]

**Debriefing works: Successful retraction of misinformation following a fake news study**

**Supplemental materials**

**Effects of time (T1/T2) and warning condition on true memory rate: the proportion of true stories for which participants reported a memory.**

| **Within Subjects Effects** | | | | | | | | | | | | | |
| --- | --- | --- | --- | --- | --- | --- | --- | --- | --- | --- | --- | --- | --- |
| **Cases** | | **Sum of Squares** | | **df** | | **Mean Square** | | **F** | | **p** | | **η²_p_** | |
| Time |  | 5.006e-4 |  | 1 |  | 5.006e-4 |  | 0.007 |  | 0.935 |  | 4.363e-6 |  |
| Time ✻ WarningCondition |  | 0.388 |  | 3 |  | 0.129 |  | 1.739 |  | 0.157 |  | 0.003 |  |
| Residuals |  | 114.724 |  | 1543 |  | 0.074 |  |  |  |  |  |  |  |
|  | | | | | | | | | | | | | |
| *Note.*  Type III Sum of Squares | | | | | | | | | | | | | |

| **Between Subjects Effects** | | | | | | | | | | | | | |
| --- | --- | --- | --- | --- | --- | --- | --- | --- | --- | --- | --- | --- | --- |
| **Cases** | | **Sum of Squares** | | **df** | | **Mean Square** | | **F** | | **p** | | **η²_p_** | |
| WarningCondition |  | 0.421 |  | 3 |  | 0.140 |  | 1.393 |  | 0.243 |  | 0.003 |  |
| Residuals |  | 155.515 |  | 1543 |  | 0.101 |  |  |  |  |  |  |  |
|  | | | | | | | | | | | | | |
| *Note.*  Type III Sum of Squares | | | | | | | | | | | | | |

| **Descriptives** | | | | | | |  |  |  |  |  |
| --- | --- | --- | --- | --- | --- | --- | --- | --- | --- | --- | --- |
| **Time** | | **WarningCondition** | | **Mean** | | **SD** | | **N** | |  |  |
| T1 |  | negative warning |  | 0.660 |  | 0.247 | |  | 401 | |  |
|  |  | no misinformation warning |  | 0.690 |  | 0.235 | |  | 377 | |  |
|  |  | no posters |  | 0.688 |  | 0.235 | |  | 383 | |  |
|  |  | positive warning |  | 0.675 |  | 0.247 | |  | 386 | |  |
| T2 |  | negative warning |  | 0.661 |  | 0.360 | |  | 401 | |  |
|  |  | no misinformation warning |  | 0.664 |  | 0.343 | |  | 377 | |  |
|  |  | no posters |  | 0.675 |  | 0.331 | |  | 383 | |  |
|  |  | positive warning |  | 0.710 |  | 0.332 | |  | 386 | |  |
|  | | | | | | |  |  |  |  |  |

**Effects of time (T1/T2), warning condition and story type (true/fake) on truthfulness ratings.**

| **Within Subjects Effects** | | | | | | | | | | | | | |
| --- | --- | --- | --- | --- | --- | --- | --- | --- | --- | --- | --- | --- | --- |
| **Cases** | | **Sum of Squares** | | **df** | | **Mean Square** | | **F** | | **p** | | **η²_p_** | |
| Time |  | 1377.96 |  | 1 |  | 1377.96 |  | 2.41 |  | 0.12 |  | 1.64e-3 |  |
| Time ✻ WarningCondition |  | 189.30 |  | 3 |  | 63.10 |  | 0.11 |  | 0.95 |  | 2.26e-4 |  |
| Residuals |  | 839191.83 |  | 1468 |  | 571.66 |  |  |  |  |  |  |  |
| Story type |  | 3.09e+6 |  | 1 |  | 3.09e+6 |  | 5859.75 |  | < .001 |  | 0.80 |  |
| Story type ✻ WarningCondition |  | 1259.92 |  | 3 |  | 419.97 |  | 0.80 |  | 0.50 |  | 1.62e-3 |  |
| Residuals |  | 775204.65 |  | 1468 |  | 528.07 |  |  |  |  |  |  |  |
| Time ✻ Story type |  | 53570.15 |  | 1 |  | 53570.15 |  | 124.37 |  | < .001 |  | 0.08 |  |
| Time ✻ Story type ✻ WarningCondition |  | 264.54 |  | 3 |  | 88.18 |  | 0.20 |  | 0.89 |  | 4.18e-4 |  |
| Residuals |  | 632291.09 |  | 1468 |  | 430.72 |  |  |  |  |  |  |  |
|  | | | | | | | | | | | | | |
| *Note.*  Type III Sum of Squares | | | | | | | | | | | | | |

| **Between Subjects Effects** | | | | | | | | | | | | | |
| --- | --- | --- | --- | --- | --- | --- | --- | --- | --- | --- | --- | --- | --- |
| **Cases** | | **Sum of Squares** | | **df** | | **Mean Square** | | **F** | | **p** | | **η²_p_** | |
| WarningCondition |  | 3205.27 |  | 3 |  | 1068.42 |  | 1.75 |  | 0.16 |  | 3.55e-3 |  |
| Residuals |  | 898638.02 |  | 1468 |  | 612.15 |  |  |  |  |  |  |  |
|  | | | | | | | | | | | | | |
| *Note.*  Type III Sum of Squares | | | | | | | | | | | | | |

| **Descriptive statistics** | | | | | | | | | | | |
| --- | --- | --- | --- | --- | --- | --- | --- | --- | --- | --- | --- |
| **Time** | | **Story type** | | **WarningCondition** | | **Mean** | | **SD** | | **N** | |
| T1 |  | True |  | negative warning |  | 68.18 |  | 18.25 |  | 383 |  |
|  |  |  |  | no misinfo warning |  | 69.40 |  | 18.10 |  | 356 |  |
|  |  |  |  | no posters |  | 67.56 |  | 18.40 |  | 366 |  |
|  |  |  |  | positive warning |  | 69.45 |  | 18.01 |  | 367 |  |
|  |  | Fake |  | negative warning |  | 27.65 |  | 23.22 |  | 383 |  |
|  |  |  |  | no misinfo warning |  | 29.97 |  | 25.47 |  | 356 |  |
|  |  |  |  | no posters |  | 29.21 |  | 23.58 |  | 366 |  |
|  |  |  |  | positive warning |  | 28.44 |  | 24.57 |  | 367 |  |
| T2 |  | True |  | negative warning |  | 72.78 |  | 27.11 |  | 383 |  |
|  |  |  |  | no misinfo warning |  | 73.82 |  | 27.37 |  | 356 |  |
|  |  |  |  | no posters |  | 73.01 |  | 27.13 |  | 366 |  |
|  |  |  |  | positive warning |  | 75.24 |  | 28.14 |  | 367 |  |
|  |  | Fake |  | negative warning |  | 20.63 |  | 20.52 |  | 383 |  |
|  |  |  |  | no misinfo warning |  | 23.36 |  | 21.73 |  | 356 |  |
|  |  |  |  | no posters |  | 21.33 |  | 21.09 |  | 366 |  |
|  |  |  |  | positive warning |  | 21.94 |  | 23.58 |  | 367 |  |
|  | | | | | | | | | | | |

**Effects of false memory for fake stories at Time1 and warning condition on related behavioural intentions after one week.**

1. Intention to eat more spicy food at Time 2, as a function of original false memory for the chilli peppers story and warning condition:

| **ANOVA** | | | | | | | | | | | | | |
| --- | --- | --- | --- | --- | --- | --- | --- | --- | --- | --- | --- | --- | --- |
| **Cases** | | **Sum of Squares** | | **df** | | **Mean Square** | | **F** | | **p** | | **η² _p_** | |
| Chili_FalseMem_T1 |  | 0.88 |  | 1 |  | 0.88 |  | 0.47 |  | 0.49 |  | 6.19e -4 |  |
| WarningCondition |  | 2.07 |  | 3 |  | 0.69 |  | 0.37 |  | 0.77 |  | 1.46e -3 |  |
| WarningCondition ✻ Chili_FalseMem_T1 |  | 3.02 |  | 3 |  | 1.01 |  | 0.54 |  | 0.66 |  | 2.12e -3 |  |
| Residuals |  | 1418.54 |  | 761 |  | 1.86 |  |  |  |  |  |  |  |
|  | | | | | | | | | | | | | |
| *Note.*  Type III Sum of Squares | | | | | | | | | | | | | |

| **Descriptive statistics** | | | | | | | | |  |
| --- | --- | --- | --- | --- | --- | --- | --- | --- | --- |
| **Chili_FalseMemory_T1** | | **Warning Condition** | | **Mean** | | **SD** | | **N** |  |
| memory |  | negative warning |  | 2.38 |  | 1.06 |  | 8 |  |
|  |  | no misinformation warning |  | 3.00 |  | 1.26 |  | 11 |  |
|  |  | no posters |  | 2.90 |  | 1.62 |  | 20 |  |
|  |  | positive warning |  | 2.70 |  | 1.64 |  | 10 |  |
| no memory |  | negative warning |  | 2.73 |  | 1.40 |  | 206 |  |
|  |  | no misinformation warning |  | 2.67 |  | 1.37 |  | 173 |  |
|  |  | no posters |  | 2.55 |  | 1.33 |  | 186 |  |
|  |  | positive warning |  | 2.44 |  | 1.32 |  | 155 |  |
|  | | | | | | | | |  |

1. Intention to drink more coffee at Time 2, as a function of original false memory for the coffee story and warning condition:

| **ANOVA** | | | | | | | | | | | | | |
| --- | --- | --- | --- | --- | --- | --- | --- | --- | --- | --- | --- | --- | --- |
| **Cases** | | **Sum of Squares** | | **df** | | **Mean Square** | | **F** | | **p** | | **η² _p_** | |
| Coffee_FalseMemory_T1 |  | 3.44e -4 |  | 1 |  | 3.44e -4 |  | 1.87e -4 |  | 0.99 |  | 2.46e -7 |  |
| WarningCondition |  | 1.74 |  | 3 |  | 0.58 |  | 0.31 |  | 0.81 |  | 1.24e -3 |  |
| Coffee_FalseMem_T1 ✻ WarningCondition |  | 2.15 |  | 3 |  | 0.72 |  | 0.39 |  | 0.76 |  | 1.53e -3 |  |
| Residuals |  | 1401.74 |  | 762 |  | 1.84 |  |  |  |  |  |  |  |
|  | | | | | | | | | | | | | |
| Note.  Type III Sum of Squares | | | | | | | | | | | | | |

| **Descriptive statistics** | | | | | | | | |  |
| --- | --- | --- | --- | --- | --- | --- | --- | --- | --- |
| **Coffee_FalseMemory_T1** | | **Warning Condition** | | **Mean** | | **SD** | | **N** |  |
| memory |  | negative warning |  | 2.59 |  | 1.37 |  | 27 |  |
|  |  | no misinformation warning |  | 2.21 |  | 1.40 |  | 19 |  |
|  |  | no posters |  | 2.38 |  | 1.28 |  | 21 |  |
|  |  | positive warning |  | 2.59 |  | 1.30 |  | 22 |  |
| no memory |  | negative warning |  | 2.42 |  | 1.39 |  | 160 |  |
|  |  | no misinformation warning |  | 2.46 |  | 1.30 |  | 177 |  |
|  |  | no posters |  | 2.43 |  | 1.42 |  | 175 |  |
|  |  | positive warning |  | 2.45 |  | 1.33 |  | 169 |  |
|  | | | | | | | | |  |

1. Intention to get a COVID-19 vaccine at Time 2, as a function of original false memory for the vaccine story and warning condition:

| **ANOVA** | | | | | | | | | | | | | |
| --- | --- | --- | --- | --- | --- | --- | --- | --- | --- | --- | --- | --- | --- |
| **Cases** | | **Sum of Squares** | | **df** | | **Mean Square** | | **F** | | **p** | | **η² _p_** | |
| Vaccine_FalseMem_T1 |  | 6.18 |  | 1 |  | 6.18 |  | 2.19 |  | 0.14 |  | 2.88e -3 |  |
| WarningCondition |  | 2.38 |  | 3 |  | 0.79 |  | 0.28 |  | 0.84 |  | 1.11e -3 |  |
| WarningCondition ✻ Vaccine_FalseMem_T1 |  | 6.61 |  | 3 |  | 2.20 |  | 0.78 |  | 0.50 |  | 3.08e -3 |  |
| Residuals |  | 2142.15 |  | 759 |  | 2.82 |  |  |  |  |  |  |  |
|  | | | | | | | | | | | | | |
| *Note.*  Type III Sum of Squares | | | | | | | | | | | | | |

| **Descriptive statistics** | | | | | | | | | |
| --- | --- | --- | --- | --- | --- | --- | --- | --- | --- |
| **Vaccine_FalseMem_T1** | | **Warning Condition** | **Mean** | | | **SD** | | **N** | |
| memory |  | negative warning |  | 5.21 |  | 1.97 |  | 14 |  |
|  |  | no misinformation warning |  | 4.93 |  | 1.98 |  | 14 |  |
|  |  | no posters |  | 4.92 |  | 2.02 |  | 13 |  |
|  |  | positive warning |  | 5.59 |  | 1.66 |  | 17 |  |
| no memory |  | negative warning |  | 5.50 |  | 1.57 |  | 191 |  |
|  |  | no misinformation warning |  | 5.58 |  | 1.66 |  | 166 |  |
|  |  | no posters |  | 5.54 |  | 1.63 |  | 162 |  |
|  |  | positive warning |  | 5.39 |  | 1.78 |  | 190 |  |
|  | | | | | | | | | |

1. Intention to download a contact tracing app at Time 2, as a function of original false memory for the contact tracing story and warning condition:

| **ANOVA** | | | | | | | | | | | | | |
| --- | --- | --- | --- | --- | --- | --- | --- | --- | --- | --- | --- | --- | --- |
| **Cases** | | **Sum of Squares** | | **df** | | **Mean Square** | | **F** | | **p** | | **η² _p_** | |
| ContactTracing_FalseMem_T1 |  | 1.91 |  | 1 |  | 1.91 |  | 0.49 |  | 0.48 |  | 6.45e -4 |  |
| WarningCondition |  | 9.82 |  | 3 |  | 3.27 |  | 0.84 |  | 0.47 |  | 3.31e -3 |  |
| ContactTracing_FalseMem_T1 ✻ WarningCondition |  | 5.28 |  | 3 |  | 1.76 |  | 0.45 |  | 0.72 |  | 1.78e -3 |  |
| Residuals |  | 2955.70 |  | 761 |  | 3.88 |  |  |  |  |  |  |  |
|  | | | | | | | | | | | | | |
| *Note.*  Type III Sum of Squares | | | | | | | | | | | | | |

| **Descriptive statistics** | | | | | | | | |  |
| --- | --- | --- | --- | --- | --- | --- | --- | --- | --- |
| **ContactTracing_FalseMem_T1** | | **Warning Condition** | | **Mean** | | **SD** | | **N** |  |
| memory |  | negative warning |  | 4.42 |  | 2.07 |  | 50 |  |
|  |  | no misinformation warning |  | 4.89 |  | 2.28 |  | 37 |  |
|  |  | no posters |  | 4.96 |  | 1.95 |  | 45 |  |
|  |  | positive warning |  | 4.89 |  | 2.15 |  | 46 |  |
| no memory |  | negative warning |  | 4.84 |  | 1.84 |  | 140 |  |
|  |  | no misinformation warning |  | 4.91 |  | 1.98 |  | 153 |  |
|  |  | no posters |  | 4.85 |  | 2.01 |  | 141 |  |
|  |  | positive warning |  | 5.04 |  | 1.88 |  | 157 |  |
|  | | | | | | | | |  |
